# Mineral composition and phosphorus digestibility in feed phosphates fed to pigs and poultry

**DOI:** 10.5713/ab.22.0322

**Published:** 2022-11-14

**Authors:** Su A Lee, Diego A. Lopez, Hans H. Stein

**Affiliations:** 1 Department of Animal Sciences, University of Illinois, Urbana, IL 61801, USA; 2 Current address: Kansas State University, Manhattan, Kansas, KS 66506, USA

**Keywords:** Digestibility, Feed Phosphate, Impurity, Mineral, Pig, Poultry

## Abstract

Phosphorus (P) is a macro mineral needed for bone mineralization and cell membrane structure and P is also involved in several fundamental pathways of metabolism in the body. Because of the low concentration and digestibility of P in plant ingredients that are the main components of diets for poultry and pigs, feed phosphates are usually included in diets in addition to the P contributed by plant ingredients. The most widely used feed phosphates in poultry and swine diets are dicalcium phosphate (DCP) and monocalcium phosphate (MCP), but tricalcium phosphate (TCP), monosodium phosphate (MSP), and magnesium phosphate (MgP) may be used as well. Because feed phosphates are mostly produced from rock phosphate, feed phosphates have impurities that contain minerals other than P. Concentrations of P in feed phosphates range from 14.8% (MgP) to 25.7% (MSP). The standardized total tract digestibility (STTD) of P in pigs ranges from 71% (TCP) to 95% (MSP). The STTD of Ca and the standardized ileal digestibility (SID) of P and Ca in feed phosphates fed to pigs and poultry have been determined only in a few experiments. Available data indicate that the STTD of Ca and SID of P in MCP are greater than in DCP in both poultry and pigs, but the SID of Ca is similar between DCP and MCP fed to broilers. Information on mineral concentrations and digestibility values in feed phosphates is needed in diet formulation for pigs and poultry, but if diets are formulated to contain equal concentrations of digestible P and Ca, it is unlikely that animal performance will be impacted by the source of feed phosphates used in the diet.

## INTRODUCTION

Phosphorus (P) is a macro mineral needed for bone mineralization and cell membrane structure and P is also involved in several fundamental pathways of metabolism in the body. Phosphorus nutrition has been studied more intensely than the nutrition of any other mineral due to its importance in nutrition, high cost, and potential for contributing to pollution of the external environment [1].

Dietary P can be provided by feed ingredients of plant or animal origin. Plant ingredients used in poultry and swine nutrition are mainly grains and co-products from oilseeds, but grain co-products are also used. The concentration of P in cereal grains ranges from 0.18% (polished rice) to 0.38% (triticale), whereas for grain co-products, P concentration ranges from 0.24% (corn gluten meal) to 2.58% (defatted rice bran), and for oilseed meals from 0.52% (palm kernel expellers) to 1.22% (dehulled sunflower meal) [2]. However, up to 92% of total P in plant feed ingredients is phytate-bound [2–4], which results in low utilization of P from plants by poultry and pigs [5,6]. Fish meal, meat and bone meal, blood meal, and co-products of milk are the main animal-origin ingredients that are used in poultry and swine diets [7–9]. Because P in animal-origin ingredients is not bound to phytate, it is highly digestible to pigs whereas P from plants generally has low digestibility [1,5]. However, animal-origin ingredients are mostly used for weanling pigs, whereas diets for growing-finishing pigs, sows, and poultry primarily contain plant ingredients.

Because of the low concentration and digestibility of P in plant ingredients, feed phosphates are usually included in diets for pigs and poultry in addition to the P contributed by plant- and animal-origin ingredients [10]. Mineral concentrations, digestibility values, and physicochemical characteristics of feed phosphates are therefore of importance to the poultry and swine feed industry. However, summarized data on characteristics and P digestibility in feed phosphates fed to poultry and pigs are limited. Therefore, the objective of the current work was to review current knowledge about feed phosphates used in the poultry and swine feed industries and to summarize data on P digestibility in feed phosphates fed to pigs and poultry.

## FEED PHOSPHATES USED IN POULTRY AND SWINE NUTRITION

The most widely used feed phosphates are dicalcium phosphate (DCP) and monocalcium phosphate (MCP) [11], but tricalcium phosphate (TCP), monosodium phosphate (MSP), and magnesium phosphate (MgP) may be used as well [12–14].

Concentrations of P in feed phosphates range from 14.8% (MgP) to 25.7% (MSP; Table 1). The concentration of dry matter (DM) in DCP, MCP, MgP, and MSP is greater than 90% and the concentration of ash is greater than 78%. The difference between DM and ash is a result of the loss of crystalline water, carbon dioxide, and volatile minerals during the ashing procedure [12,15]. Crystalline water originates from some of the phosphate salts in feed phosphates, whereas carbon dioxide is lost from carbonates that usually also are present in feed phosphates.

### Dicalcium phosphate and monocalcium phosphate

**Figure 1:**
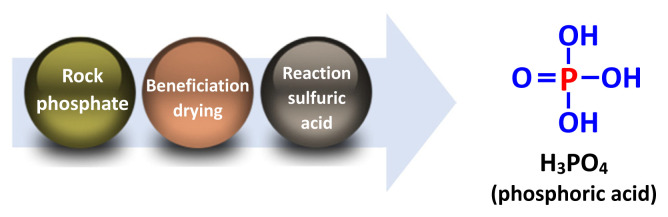
Production of phosphoric acid.

**Figure 2:**
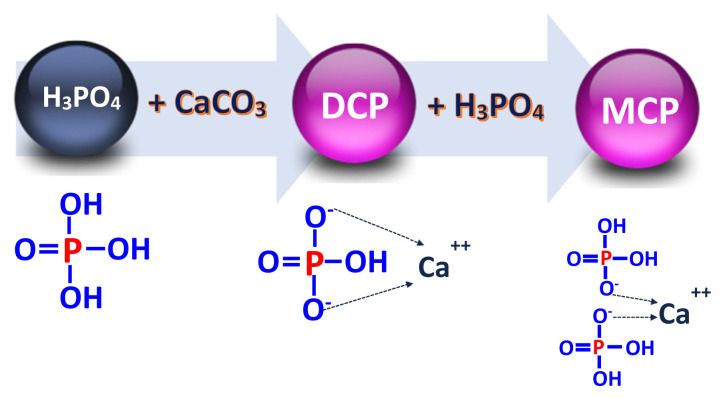
Production of DCP and MCP. DCP, dicalcium phosphate; MCP, monocalcium phosphate.

Feed phosphates are products of the wet processing crushing of phosphate rock from volcanic or sedimentary origin. Phosphorus is extracted from the rock and released in the form of phosphoric acid (H_3_PO_4_) after reaction of the rock with sulfuric acid although hydrochloric acid may also be used (Figure 1) [16,17]. A second reaction in which phosphoric acid is reacted with calcium carbonate (CaCO_3_) results in the production of DCP (CaHPO_4_) and MCP [Ca(H_2_PO_4_)_2_; Figure 2] [18]:


$$\begin{array}{l} \text{DCP:}\;{\text{H}}_{3}{\text{PO}}_{4}+{\text{CaCO}}_{3}\to {\text{H}}_{2}\text{O}+{\text{CO}}_{2}+{\text{CaHPO}}_{4},\\ \text{MCP:}\;2({\text{H}}_{3}{\text{PO}}_{4})+{\text{CaCO}}_{3}\to {\text{H}}_{2}\text{O}+{\text{CO}}_{2}+\text{Ca}{\left({\text{H}}_{2}{\text{PO}}_{4}\right)}_{2}. \end{array}$$

The reaction of phosphoric acid with calcium carbonate will naturally reach a chemical equilibrium that results in a mixture of DCP and MCP [19,20]. In commercial sources of calcium phosphates that are produced in the U.S., Ca concentrations are more variable among different sources compared with concentrations of P because the reaction between phosphoric acid and calcium carbonate is stopped according to the amount of total P desired in the final product. Producers of DCP and MCP have to guarantee a minimum concentration of P in the final products, which is controlled by the amount of phosphoric acid that is added to calcium carbonate. The reaction is usually stopped at 18.5% P to produce DCP, but the reaction continues until the product contains 21.0% P if MCP is produced. Therefore, final products have a relatively constant concentration of P, but variations in Ca concentrations are often observed. However, because the production of DCP and MCP is a continuous process, feed phosphates that are sold as DCP or MCP usually contain both DCP and MCP and the only difference is that there is less DCP in a product designated as MCP than if the product is designated as DCP [19,21]. Dicalcium phosphate can be present in both anhydrate (CaHPO_4_) and hydrate forms (CaHPO_4_·H_2_O or CaHPO_4_·2H_2_O), but MCP exists mainly in a monohydrate form [Ca(H_2_PO_4_)_2_·H_2_O]. Neutralization of phosphoric acids with calcium carbonate results in a slurry that contains DCP in the hydrated form, but heating at 65°C to 70°C is needed to dry the slurry, which results in some of the hydrated DCP becoming anhydrated DCP (CaHPO_4_). In commercial DCP, approximately 35% is in the dihydrated form (Table 2).

In pure sources of DCP (molecular weight = 136.1 g/mol) and MCP (molecular weight = 234.05 g/mol), concentrations of P are 22.8% and 26.5%, respectively, and concentrations of Ca are 29.5% and 17.1%, respectively. However, feed grade sources of these ingredients have lower concentrations of P and Ca. The reason is that minerals other than Ca and P are present in feed grade phosphates along with unreacted calcium carbonate. Therefore, although the process of producing feed grade MCP and DCP is designed to eliminate impurities that may be harmful to animals, other minerals are usually present in feed phosphates, which is often due to impurities in the calcium carbonate that is used in the production process. Some of the minerals considered impurities in feed phosphate such as Mg, S, Fe, Al, and Na can form phosphate salts including magnesium phosphate [Mg(H_2_PO_4_)_2_·4H_2_O], calcium sulfate (CaSO_4_·H_2_O), ferrous phosphate (FePO_4_·2H_2_O), aluminum phosphate (AlPO_4_), and others [19]. Therefore, the calcium phosphates typically used in the feed industry contain several minerals other than P and Ca.

### Tricalcium phosphate

Tricalcium phosphate [Ca_3_(PO_4_)_2_] is produced by reacting phosphoric acid with calcium carbonate to form calcium dihydrogen phosphite [Ca(H_2_PO_3_)_2_] followed by calcination above 900°C:


$$\text{Ca}{\left({\text{H}}_{2}{\text{PO}}_{3}\right)}_{2}+2\text{Ca}{\left(\text{OH}\right)}_{2}+\text{natural gas}\to (\text{calcination})\to {\text{Ca}}_{3}{\left({\text{PO}}_{4}\right)}_{2}.$$

When the phosphoric acid is neutralized, calcium phos phate hydroxyapatite, Ca_10_(PO_4_)_6_(OH)_2_, is also formed [22,23]. The pure forms of TCP and hydroxyapatite are not used in animal feeds, but defluorinated rock phosphate, which is commercially available, is known as feed grade TCP because it mainly contains TCP [12]. By applying the high temperature during the calcination process, sulfur or fluorine that are considered harmful to animals are mostly removed [24], but some Na from the original rock remains in the final product (<5.5%) [12]. Feed-grade TCP is used in poultry diets, but it is not frequently included in diets for pigs in North America. However, some countries in Asia use TCP as source of Ca and P in diets for pigs.

### Monosodium phosphate

Monosodium dihydrogen phosphate is produced from the reaction of phosphoric acid and sodium hydroxide or carbonate [20]. The reaction between phosphoric acid and sodium hydroxide (NaOH) or sodium carbonate (Na_2_CO_3_) is the initial reaction to produce MSP and depending on the manufacturer, different reagents are used:


$$\begin{array}{l} \text{Using NaOH:}\;{\text{H}}_{3}{\text{PO}}_{4}+3\text{NaOH}\to {\text{Na}}_{3}{\text{PO}}_{4}+3{\text{H}}_{2}\text{O}\\ \text{Using }{\text{Na}}_{2}{\text{CO}}_{3}:\;2{\text{H}}_{3}{\text{PO}}_{4}+3{\text{Na}}_{2}{\text{CO}}_{3}\to 2{\text{Na}}_{3}{\text{PO}}_{4}+3{\text{H}}_{2}{\text{CO}}_{3}. \end{array}$$

**Figure 3:**
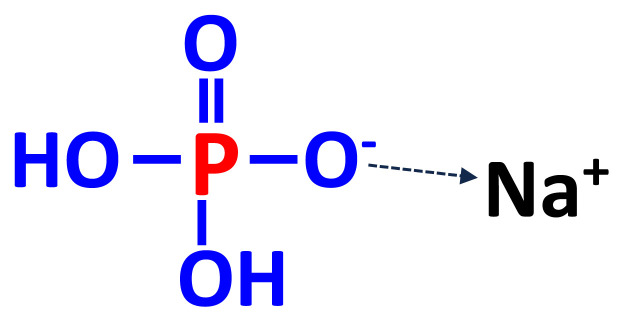
Chemical structures of MSP (NaH_2_PO_4_). monosodium phosphate; MSP, monosodium phosphate.

The end product of the initial reactions is trisodium phos phate (Na_3_PO_4_), but treatment with water in scrubbers results in the production of monosodium dihydrogen phosphate (NaH_2_PO_4_; Figure 3) [25].

The concentration of P in feed grade MSP is greater than 24% [3]. Requirements for Na can be met by the inclusion of salt in the diets, which also will result in Cl meeting the requirement [3], and MSP is, therefore, rarely used as a source of Na in practical diets for pigs. However, MSP is sometimes used in research diets for pigs and because of the high digestibility of P in MSP, it is often used as the standard to estimate the relative bioavailability of P in different feed ingredients [11,26,27].

### Magnesium phosphate

**Figure 4:**
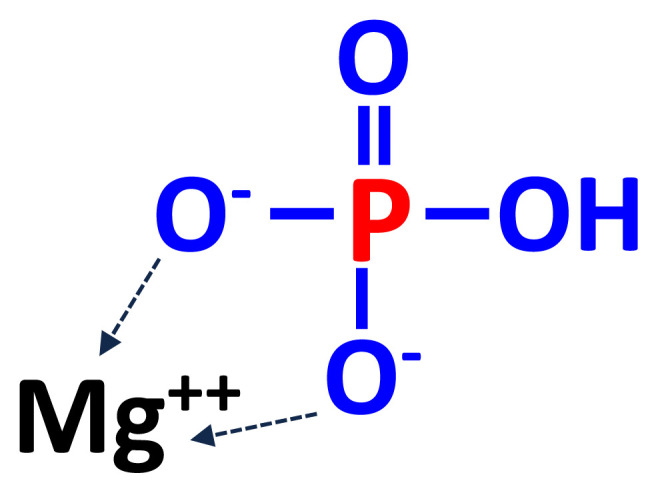
Chemical structures of MgP (MgHPO4). MgP, magnesium phosphate.

Magnesium phosphate (MgHPO_4_) may be produced by a double decomposition reaction between disodium phosphate and magnesium salts or by neutralizing solutions containing magnesium salts and phosphoric acids with caustic soda (NaOH; Figure 4) [22]. Most MgP salts are in hydrated forms (MgHPO_4_·H_2_O). Commercial MgP often has a greater concentration of S than other feed phosphates, but even if MgP is used to provide the majority of P in diets, the concentration of S will be less than the concentration that is expected to negatively affect growth of pigs due to the low inclusion of feed phosphates in the final diets and the relatively low absorption of S in the pig [3,28]. Magnesium is usually not added to practical diets for pigs, but MgP is sometimes used in mineral premixes if feed ingredients with low availability of Mg are used [3]. Magnesium phosphate is also used in animal nutrition, especially in ruminants, because deficiency of Mg is more common in forage-based diets for ruminant animals. The trihydrate form [MgHPO_4_·3(H_2_O)] is the only stable form at 25°C.

## DIGESTIBILITY OF P IN FEED PHOSPHATES

### Determination of digestibility of P in pigs and poultry

Historically, values for the relative bioavailability of P in feed ingredients were generated using MSP or MCP as the standard [29] and these values were used to formulate diets for pigs [30]. However, values for the relative bioavailability of P are not always additive in mixed diets, and values vary depending on the digestibility of P in the standard [11]. It was, therefore, recognized that formulating diets based on values for digestible P is more accurate than using values for the relative bioavailability of P [3].

Because P is mostly absorbed before the end of the small intestine, there is no difference between values for ileal digestibility and total tract digestibility of P [31–33], although some hindgut disappearance of P in pigs has been reported ([34–36]). Because it is easier and less expensive to measure total tract digestibility of P than ileal digestibility of P, total tract digestibility of P is usually measured. By correcting the apparent total tract digestibility of P for the basal endogenous losses of P, values for the standardized total tract digestibility (STTD) of P are calculated. Values for the STTD of P are not influenced by the concentration of P in the diet and those values are, therefore, not underestimated if the concentration of P in the diet is low [37]. Values for the STTD of P are also additive in a mixed diet [38,39], which is a prerequisite for accurate diet formulation. It is, therefore, recommended that diets for pigs are formulated based on the STTD of P in individual ingredients [3].

Values for total tract digestibility of P in poultry are diffi cult to obtain because the excreta of chickens contains both fecal and urine excretions. Therefore, ileal digestibility of P in feed ingredients fed to poultry may be determined to exclude urinary excretion in the excreta [40]. Because values for standardized ileal digestibility (SID) of P are additive [41] and are not affected by dietary P [40], the SID of P in various feed ingredients fed to broilers has been determined. However, information on the SID of P in feed phosphates fed to poultry is limited.

### STTD and SID of P in feed phosphates fed to pigs and broiler chickens

**Figure 5:**
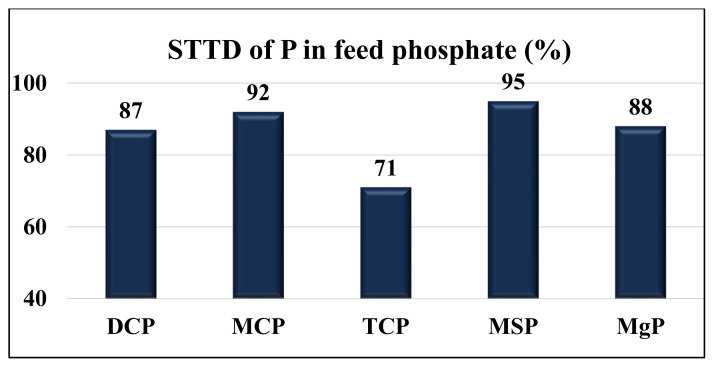
Standardized total tract digestibility (STTD) of P (%) in feed phosphate fed to pigs. DCP, dicalcium phosphate; MCP, monocalcium phosphate; TCP, tricalcium phosphate; MSP, monosodium phosphate; MgP, magnesium phosphate. Data from Petersen and Stein [21]; NRC [3]; Baker et al [54]; Kwon and Kim [48]; and Lopez [49].

Values for the STTD of P vary among different feed phosphates fed to pigs (Figure 5). Among calcium phosphates, the STTD of P in MCP (93%) is the greatest, followed by DCP (89%) and TCP (71%). The SID of P in feed phosphates has been determined only in one experiment. Among DCP, MCP, and TCP, the SID of P in MCP (89.3%) is the greatest, followed by DCP (79.5%) and TCP (56.7%) if the feed phosphates are fed to broilers chickens [42].

It appears that P in a calcium phosphate is better digested and absorbed if the calcium phosphate contains less Ca. This may be a result of the interaction between dietary Ca and P, which forms an indigestible Ca-P complex that precipitates in the intestinal tract of pigs [43,44], but more research is needed to confirm this hypothesis. Most commercial DCP is in the anhydrous form, but (di-)hydration of P molecules may increase the digestibility of P in DCP fed to pigs, because hydrated DCP is more soluble in the intestinal tracts, and thus has a greater digestibility, than the anhydrous form [5, 10,45–47].

The STTD of P in MgP (88%) fed to pigs is less than in MCP and MSP, but greater compared with TCP. The STTD of P in MSP is greater than in calcium phosphates or MgP [3,21,48,49]. This observation is likely the reason MSP was often used as the standard in experiments conducted to determine the relative bioavailability of P in feed ingredients.

### STTD and SID of Ca in DCP and MCP fed to pigs and broiler chickens

The STTD of Ca in feed ingredients has been determined because digestible Ca is more additive in mixed diets if values are corrected with endogenous losses [50]. Use of exogenous phytase may increase the STTD of Ca in calcium carbonate and some other feed ingredients, but that is not the case for the STTD of Ca in MCP and DCP [51,52]. However, the STTD of Ca in feed phosphates has been determined only in a few experiments. The STTD of Ca in MCP (86%) is likely greater than in DCP (77%) [51]. However, because of the greater concentration of Ca in DCP than in MCP, the concentration of standardized total tract digestible Ca in DCP is close to that in MCP. Variations in the STTD of Ca among different sources of DCP and MCP appear to be low [52].

The SID by broiler chickens of Ca in DCP and MCP was summarized by Walk et al [53], although not many experiments have determined the SID of Ca in feed phosphates fed to poultry. The SID of Ca in both DCP and MCP fed to broilers is 36%, which is much lower compared with pigs.

## CONCLUSION

The current contribution discussed how feed phosphates are produced, how much P and other minerals are included in each feed phosphate, and how much P is utilized if they are fed to pigs and poultry. Production of feed phosphates has been designed to meet a minimum concentration of P using phosphate rock, which results in variations in concentrations of other minerals. Feed phosphate sources contain 15% to 26% P and values for the STTD of P vary with different feed phosphates. Information on both mineral concentrations and digestibility values in feed phosphates is needed in diet formulation for pigs and poultry because each source contains different concentrations of digestible P. However, if diets are formulated to contain equal concentrations of digestible P and Ca, it is unlikely that animal performance will be impacted by the source of feed phosphates used in the diet.

## Figures and Tables

**Table 1:** Concentrations of macro- and micro-minerals in feed phosphates^1)^

Item	DCP	MCP	TCP	MSP	MgP
Dry matter (%)	94.9±0.60	93.0±0.78	-	99.2±0.41	96.6
Ash (%)	83.3±1.41	79.6±0.62	-	88.3±3.03	86.4
Macro minerals (%)					
Ca	21.3±2.40	16.7±0.44	34.2	0.7±0.45	1.0
P	19.2±0.28	21.9±0.86	17.7	25.7±1.95	14.8
Mg	0.7±0.51	0.7±0.42	0.4	<0.1	24.7
Na	<0.1	<0.1	6.0	20.5±0.00	0.7
K	0.1±0.02	0.1±0.02	-	<0.1	0.1
S	0.4±0.21	0.2±0.07	-	<0.1	1.7
Micro minerals					
Co (mg/kg)	3.1±2.21	3.0±2.21	-	<2.3	4.0
Cu (mg/kg)	7.7±6.77	8.7±7.20	-	<0.6	2.0
F (%)	0.1±0.02	0.2±0.04	-	<0.1	0.1
Fe (%)	0.7±0.50	0.6±0.44	-	<0.3	0.3
Mn (%)	0.04±0.02	0.04±0.02	-	<0.1	<0.1
Zn (mg/kg)	89.0±66.03	123.4±72.28	-	28.0±27.00	50.0
Other minerals (mg/kg)					
Al	553.4±452.59	437.1±492.14	-	1,124±456.00	161.0
As	5.7±1.48	6.0±3.26	-	1.0±0.60	1.0
Cd	3.1±2.41	3.5±2.52	-	0.3±0.08	0.2
Hg	<0.1	<0.1	-	<0.1	<0.1
Pb	0.8±0.30	1.2±1.14	-	0.1±0.04	0.2
Si	3,159±1,753	3,496±2,047	-	387.5±26.50	17,300
Free H_2_O (%)	<0.2	<0.1	-	<0.1	<0.1

DCP, dicalcium phosphate; MCP, monocalcium phosphate; TCP, tricalcium phosphate; MSP, monosodium phosphate; MgP, magnesium phosphate.

1) Information on analyzed concentrations of minerals in DCP, MCP, MSP, and MgP were from 4, 7, 2, and 1 sources, respectively [49]; values for concentrations of minerals in TCP were obtained from NRC [3].

**Table 2:** Mineral composition of commercial dicalcium phosphate (DCP) and monocalcium phosphate (MCP)^1)^

Component (%)	Chemical formula	DCP	MCP
Calcium carbonate	CaCO_3_	6.74	6.00
DCP and MCP			
MCP	Ca(H2PO_4_)_2_·H_2_O	14.19	60.98
DCP	CaHPO_4_	26.42	12.54
Dihydrated DCP	CaHPO_4_·2(H_2_O)	34.65	-
Others			
Phosphoric acid	H_3_PO_4_	0.80	1.00
Silica	SiO_2_	0.15	0.13
Calcium fluoride	CaF_2_	0.32	0.44
Sodium phosphate	NaH_2_PO_4_·2(H_2_O)	0.54	0.61
Free water	H_2_O	0.80	1.00
Aluminum phosphate	AlPO_4_	2.21	2.48
Ferrous phosphate	FePO_4_·2(H_2_O)	2.65	2.98
Calcium sulfate	CaSO_4_·H_2_O	3.51	3.95
Magnesium phosphate	Mg(H_2_PO_4_)_2_·4(H_2_O)	7.02	7.89
Total		100.00	100.00

1) Adapted from [19].
